# In Silico Identification of Genes Associated with Breast Cancer Progression and Prognosis and Novel Therapeutic Targets

**DOI:** 10.3390/biomedicines10112995

**Published:** 2022-11-21

**Authors:** Shiro Uchida, Takashi Sugino

**Affiliations:** 1 Division of Diagnostic Pathology, Kikuna Memorial Hospital, Yokohama 222-0011, Japan; 2 Pathology Division, Shizuoka Cancer Center, Shizuok 411-8777, Japan; 3 Department of Human Pathology, Juntendo University School of Medicine, Tokyo 113-8421, Japan

**Keywords:** breast cancer, tumor–node–metastasis, bioinformatics, CRISPR-Cas9, RNAi

## Abstract

Molecular mechanisms underlying breast cancer (BC) progression are complex and remain unclear. In this study, we used bioinformatic tools to identify genes associated with tumor progression mechanisms and novel therapeutic targets in BC. We identified genes with stepwise upregulated expression overlapping between the T and N stages during BC progression using LinkedOmics. We compared the expression level of each gene in BC tissues with that in normal breast tissues and evaluated differences in expression in their intrinsic subtypes and their prognostic value using UALCAN and GEPIA2. We also investigated the dependency of BC cell lines on these genes and whether they are potential therapeutic targets using DepMap. *SPDEF*, *TRIM3*, *ABCB9*, *HSPB1*, *RHBG*, *SPINT1*, *EPN3*, *LRFN2*, and *PRPH* were found to be involved in BC progression. High expression of *ABCB9* and *SPINT1* was associated with a poor prognosis. *SPDEF*, *TRIM3*, *ABCB9*, *RHBG*, *SPINT1*, and *PRPH* were found to be essential for survival in some BC cell lines (gene effect score < −0.5). *PRPH* was newly discovered to be involved in the progression of BC and the growth and survival of BC cell lines. Hence, *SPDEF*, *TRIM3*, *ABCB9*, *RHBG*, *SPINT1*, and *PRPH* may serve as novel potential therapeutic targets in BC.

## 1. Introduction

Breast cancer (BC) is the most diagnosed cancer (accounting for 30.8% of all cancers) and a leading cause of cancer-related deaths (accounting for approximately 15% of all cancers) in women [1,2]. BC is now classified according to the expression of estrogen receptor (ER), progesterone receptor (PR), and human epidermal growth factor receptor 2 (HER2) proteins, and specialized treatments for each have been shown to be available and effective. These disease-subtyping-based treatment strategies have provided more effective treatment strategies than previously available [3]. However, complete remission remains a challenge because cancers are resistant to conventional targeted therapies. Therefore, molecular elucidation of BC is needed to further improve BC outcomes [4]. The complex molecular mechanisms underlying BC progression remain unclear. Therefore, it is important to identify more accurate tumor progression and establish novel and effective therapeutic methods.

The tumor–node–metastasis (TNM) system was contrived by Pierre Denoix in the 1940s; it has become the basic principle of tumor staging in the world. Generally, local (only in the affected organ), regional (spread to nearby lymph nodes), and metastatic (spread to distant organs) cancers are recognized to have worsening prognosis. BC can be traced to this stepwise progression [5]. The TNM staging system is useful for not only determining the stage but also assessing prognosis, treatment planning, and treatment efficacy. Determining the genes involved in the progression of BC could also lead to the discovery of new therapeutic approaches that target these genes. Recently, patient samples and in vitro and in vivo analyses have shown that several novel genes promote treatment resistance and progression of triple negative BC (TNBC); these include liver x receptor alpha (*LXR alpha*) [6], semaphorin 6D (*SEMA6D*) [7], and integrin subunit alpha 7 (*ITGA7*) [8]. In the past, simultaneous discovery of multiple genes would have required very large-scale studies; however, in recent years, public databases have made it possible to identify groups of genes associated with BC [9,10,11]. Therefore, we used bioinformatics to identify genes associated with BC progression that are involved with both the T and N stages.

As a background for the development of cancer therapeutic approaches, methods using new modalities such as nucleic acid drugs and genome editing using clustered regularly interspaced short palindromic repeats (CRISPR)-Cas9 have been devised and developed [12,13]. These act on mRNAs but not on conventional low-molecular-weight compounds or antibody drugs. In the future, nucleic acid drugs are expected to achieve high therapeutic efficacy as major anticancer agents. Moreover, recently, bioinformatic tools, such as the Cancer Dependency Map (DepMap) database (https://depmap.org/portal/, accessed on 25 April 2022) [14,15], have been developed to comprehensively screen dependency genes essential for the survival and proliferation of hundreds of cell lines with various tissue origins using RNA interference (RNAi) and CRISPR-Cas9 knockout/knockdown technologies [14,15]. These can be used to explore dependency on genes that are potential candidates for therapeutic targeting. We validated whether suppressing the expression of genes involved in breast cancer progression could be a potential therapeutic target for BC using DepMap. Indeed, successful identification of novel drug target factors using DepMap has been reported [16].

In this study, we used various bioinformatic tools to identify tumor-progression-associated genes and validate whether those genes could serve as prognostic factors or novel effective therapeutic targets in BC. We identified genes stepwise expressed in the T and N stages during BC progression, of which, we selected those with expression overlap between the T and N stages. These overlapping genes were implicated in BC progression and evaluated for their prognostic value. Because regulating these genes may halt the progression of BC, we used the DepMap database to verify whether these genes are essential for survival in BC cell lines and whether they are potential therapeutic targets.

## 2. Methods

### 2.1. LinkedOmics Analysis

**Figure 1:**
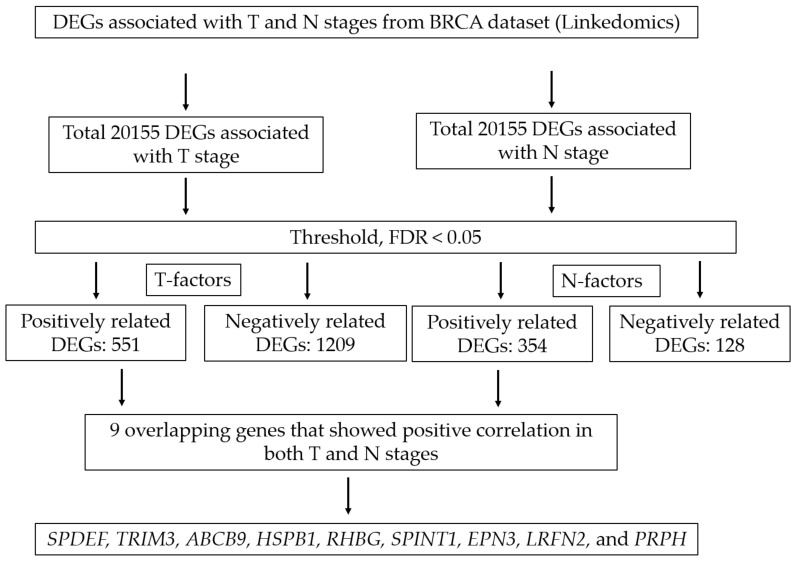
Study flowchart.

LinkedOmics (http://www.linkedomics.org/login.php) (accessed on 17 November 2022), a public online analysis tool, contains cancer-associated multidimensional datasets derived from all of the 32 The Cancer Genome Atlas (TCGA) data types [17]. We used the LinkedOmics BRCA dataset to identify differentially expressed genes (DEGs) associated with the T and N stages. The Jonckheere trend test (JT test) was performed to identify DEGs among the T (T1, T2, T3, and T4) and N stages (N0, N1, N2, and N3), and *p*-values and false discovery rate (FDR) were calculated on the web page. We then screened for DEGs that correlated with pathology_T_stage and pathology_N_stage. We adopted a statistical threshold of FDR < 0.05 and extracted genes found to be differentially expressed in the JT test and genes overlapping between the T and N stages. In both stages, 20,155 DEGs were identified, and 551 and 1209 genes were positively and negatively correlated, respectively, in the T stage, whereas 354 and 128 genes were positively and negatively correlated, respectively, in the N stage. We identified 9 genes with overlapping positive correlations in both stages. The detailed study flowchart is demonstrated in Figure 1. 

### 2.2. UALCAN Analysis

UALCAN (http://ualcan.path.uab.edu/analysis.html) (accessed on 17 November 2022), a comprehensive web resource, allows analyses based on TCGA and MET500 cohort data [18]. In our study, the expression data for the nine genes were obtained using the “Expression Analysis” module of UALCAN and the “BRCA” dataset. Student’s *t* test was used to generate *p*-values. *p*-Values were calculated and displayed on the web page.

### 2.3. Gene Expression Profiling Interactive Analysis 2 (GEPIA2) 

GEPIA2 (http://gepia2.cancer-pku.cn/) (accessed on 17 November 2022) is a web server for expression profiling and user-friendly interactive analysis based on the GTEx and TCGA databases [19]. In this study, we evaluated the prognostic value of each of the nine candidate genes. The overall survival (OS) of the patients was evaluated using the Kaplan–Meier method with a 50% (median) cut-off for both low- and high-expression groups. The log-rank test (the Mantel–Cox test) was used for hypothesis testing, and the results were displayed on the web page.

### 2.4. DepMap Analysis

DepMap (https://depmap.org/portal/) (accessed on 17 November 2022) is an accessible website [14] based on large-scale multiomics screening projects based on CRISPR-Cas9 and RNAi. Gene effect scores evaluated the effect size of either knocking out or knocking down a gene while normalizing the expression against the distribution of pan-essential and nonessential genes [14,15]. Negative scores indicated that the cell line would grow slower, whereas positive scores indicated that the cell line would grow faster after experimental manipulation. For gene effect, a score < −0.5 represents depletion in most cell lines, whereas a score < −1 represents strong killing. Therefore, we set the cut-off value at −0.5 according to the DepMap website (https://forum.depmap.org/t/depmap-genetic-dependencies-faq/131) (accessed on 17 November 2022) and a previous study [20]. We investigated the dependency on the nine genes in BC cell lines. The datasets were CRISP (DepMap 22Q1 public + score, Chronos) and RNAi (Achilles + DRIVE + Marcotte, DEMETER2), with BC subtypes including carcinoma, breast ductal carcinoma, and adenocarcinoma, with no data on cell lines. CRISP (DepMap 22Q1 public + score, Chronos) datasets were not available for Rh family B glycoprotein (*RHBG*). For *EPN3*, we did not obtain RNAi data. 

### 2.5. Statistical Analysis

The JT test was performed to detect DEGs among the T and N stages using LinkedOmics. Expression analyses between normal and tumor tissues for each gene and among intrinsic subtypes for each gene using UALCAN analysis results were performed within each web page. Statistical significance was set at *p* < 0.05.

## 3. Results

### 3.1. Screening Overlapping Upregulated DEGs

**Figure 2:**
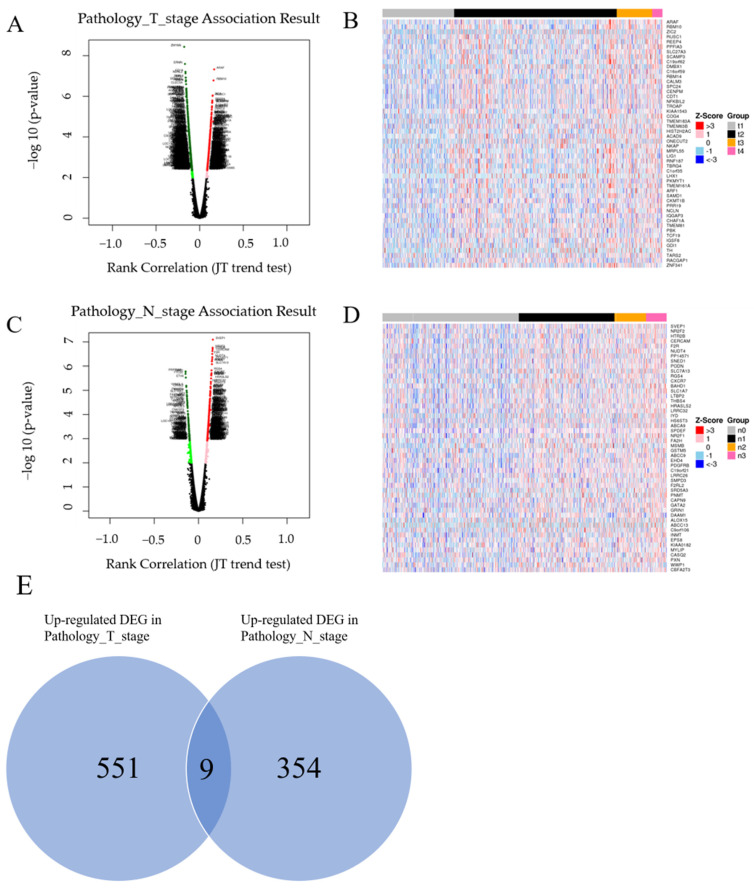
(**A**) LinkedOmics analysis. A volcano map showing genes that are positively and negatively correlated with T factor. (**B**) The heatmap shows the top 50 genes that are positively correlated with T factor. (**C**) The volcano map shows the genes that are positively and negatively correlated with N factor. (**D**) The heatmap shows the top 50 genes that are positively correlated with N factor. (**E**) Overlapping DEGs between the T (551) and N stages (354) are nine.

**Figure 3:**
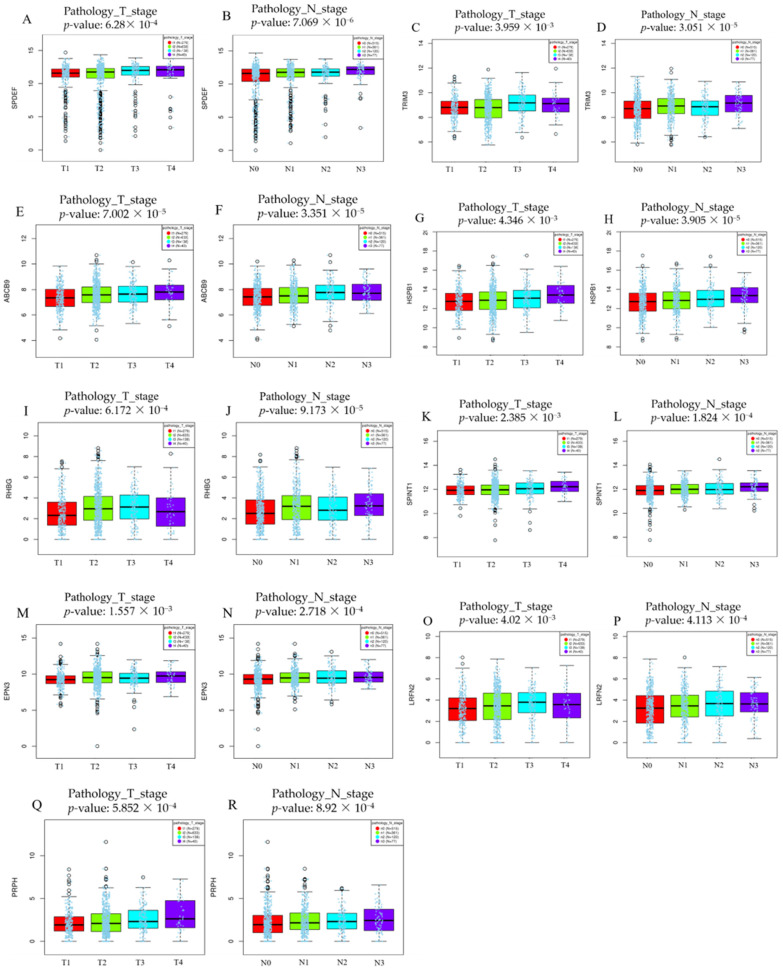
Box plot showing stepwise expression of nine genes in each T and N stage using LinkedOmics (Jonckheere trend test). As the stage progresses, the expression level also increases. (**A**) *SPDEF* (pathology_T_stage), (**B**) *SPDEF* (pathology_N_stage), (**C**) *TRIM3* (pathology_T_stage), (**D**) *TRIM3* (pathology_N_stage), (**E**) *ABCB9* (pathology_T_stage), (**F**) *ABCB9* (pathology_N_stage), (**G**) *HSPB1* (pathology_T_stage), (**H**) *HSPB1* (pathology_N_stage), (**I**) *RHBG* (pathology_T_stage), (**J**) *RHBG* (pathology_N_stage), (**K**) *SPINT1* (pathology_T_stage), (**L**) *SPINT1* (pathology_N_stage), (**M**) *EPN3* (pathology_T_stage), (**N**) *EPN3* (pathology_N_stage), (**O**) *LRFN2* (pathology_T_stage), (**P**) *LRFN2* (pathology_N_stage), (**Q**) *PRPH* (pathology_T_stage), and (**R**) *PRPH* (pathology_N_stage).

LinkedOmics analysis results are shown as a volcano plot (Figure 2A,B), and heatmaps showed the top 50 genes positively related to the T and N factors (Figure 2C,D); 551 and 354 upregulated DEGs fulfilling the positive correlation and threshold (FDR < 0.05) were identified in the T and N stages, respectively. The following nine genes overlapping between the T (551 genes) and N (354 genes) stages were identified: SAM pointed domain-containing ETS transcription factor (*SPDEF)*, tripartite motif containing 3 (*TRIM3)*, ATP binding cassette subfamily B member 9 (*ABCB9)*, heat shock protein family B (small) member 1 (*HSPB1)*, *RHBG*, serine peptidase inhibitor, Kunitz type 1 (*SPINT1)*, epsin 3 (*EPN3)*, leucine rich repeat and fibronectin type III domain-containing 2 (*LRFN2)*, and peripherin *(PRPH).* All of these genes were upregulated in both the T and N stages (Figure 2E). The details and *p*-values of these nine genes are given in Table 1. The box plot of stepwise gene expression among T and N stages is shown in Figure 3.

### 3.2. Validation of the Expression of Selected Genes in BC

**Figure 4:**
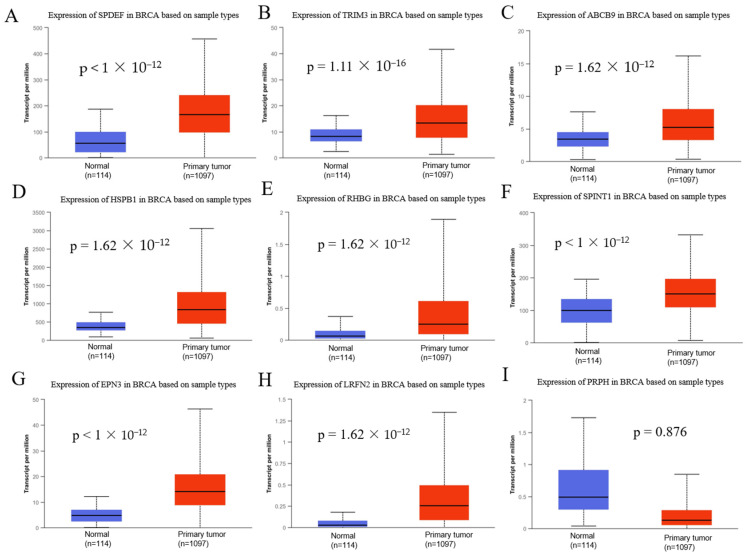
Validation of the expression of nine candidate genes in TCGA_BRCA samples and normal tissues using UALCAN. (**A**) *SPDEF*, (**B**) *TRIM3*, (**C**) *ABCB9*, (**D**) *HSPB1*, (**E**) *RHBG*, (**F**) *SPINT1*, (**G**) *EPN3*, (**H**) *LRFN2,* and (**I**) *PRPH*.

We used TCGA data from UALCAN and analyzed the expression of the nine selected genes in TCGA_BRCA (*n* = 1097) and normal tissues (*n* = 114). *SPDEF*, *TRIM3*, *ABCB9*, *HSPB1*, *RHBG*, *SPINT1*, *EPN3*, and *LRFN2* were highly expressed in BRCA tissues, and the differences were statistically significant (Figure 4A–H, *p* < 0.01). However, *PRPH* expression was lower than that in normal tissues, and the difference was not statistically significant (Figure 4I, *p* = 0.876). 

### 3.3. Validation of the Expression of Selected Genes among Intrinsic Subtypes of BRCA

**Figure 5:**
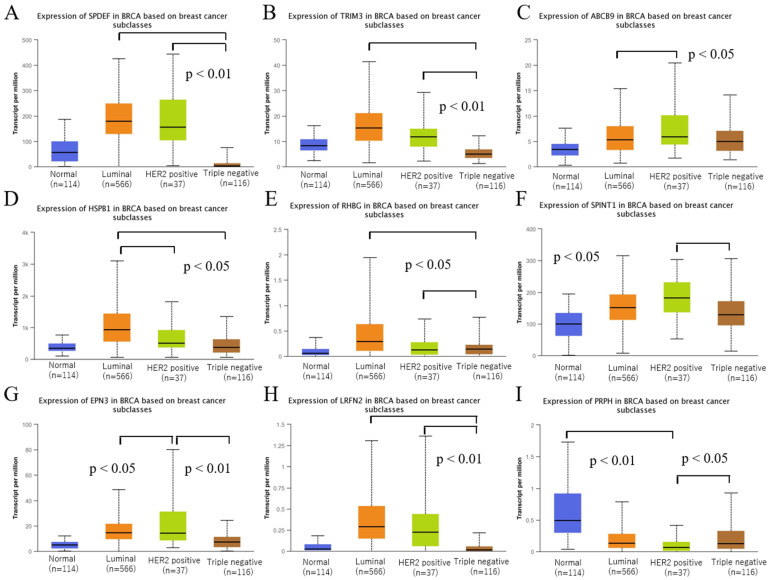
Validation of the expression of genes in the intrinsic subtype of TCGA_BRCA in UALCAN. (**A**) *SPDEF*, (**B**) *TRIM3*, (**C**) *ABCB9*, (**D**) *HSPB1*, (**E**) *RHBG*, (**F**) *SPINT1*, (**G**) *EPN3*, (**H**) *LRFN2*, and (**I**) *PRPH*.

BC is classified into three subtypes: luminal, HER2, and TNBC. *SPDEF*, *TRIM3*, and *LRFN2* expression was significantly higher in the luminal and HER2 types than in the TNBC type (*p* < 0.01, Figure 5A,B,H). *ABCB9* and *EPN3* expression was significantly higher in the HER2 type than in the luminal type (*p* < 0.05, Figure 5C,G). In the luminal type, *HSPB1* and *RHBG* expression was significantly higher than that in the HER2 and TNBC types (*p* < 0.05, Figure 5D,E). *SPINT1* expression was significantly higher in the HER2 type than in the TNBC type (*p* < 0.05, Figure 5F). *PRPH* expression was lower in all subtypes than in the normal tissues (Figure 5I). Detailed statistical analysis results are given in Supplementary Materials.

### 3.4. Survival Analysis for the Nine Genes

**Figure 6:**
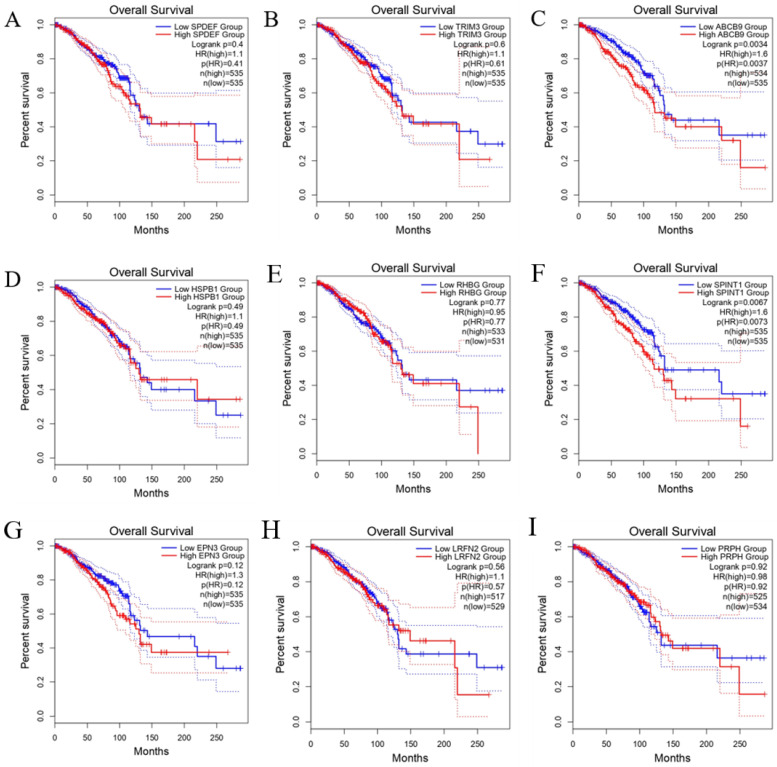
Overall survival (OS) validation of TCGA BRCA patients (GEPIA2). (**A**) *SPDEF*, (**B**) *TRIM3*, (**C**) *ABCB9*, (**D**) *HSPB1*, (**E**) *RHBG*, (**F**) *SPINT1*, (**G**) *EPN3*, (**H**) *LRFN2*, and (**I**) *PRPH*.

To investigate the prognostic value of the nine genes, we used the GEPIA2 platform (Figure 6). *ABCB9* (Figure 6C) and *SPINT1* (Figure 6F) showed significantly worse prognoses (*p* = 0.0034 and *p* = 0.0067, respectively). The expressions of *SPDEF*, *TRIM3*, *HSPB1*, *RHBG*, *EPN3*, *LRFN2*, and *PRPH* were not significant (*p* = 0.4, *p* = 0.6, *p* = 0.49, *p* = 0.77, *p* = 0.12, *p* = 0.56, and *p* = 0.92, respectively). 

### 3.5. CRISPR-Cas9 and RNAi Screening Using DepMap 

**Figure 7:**
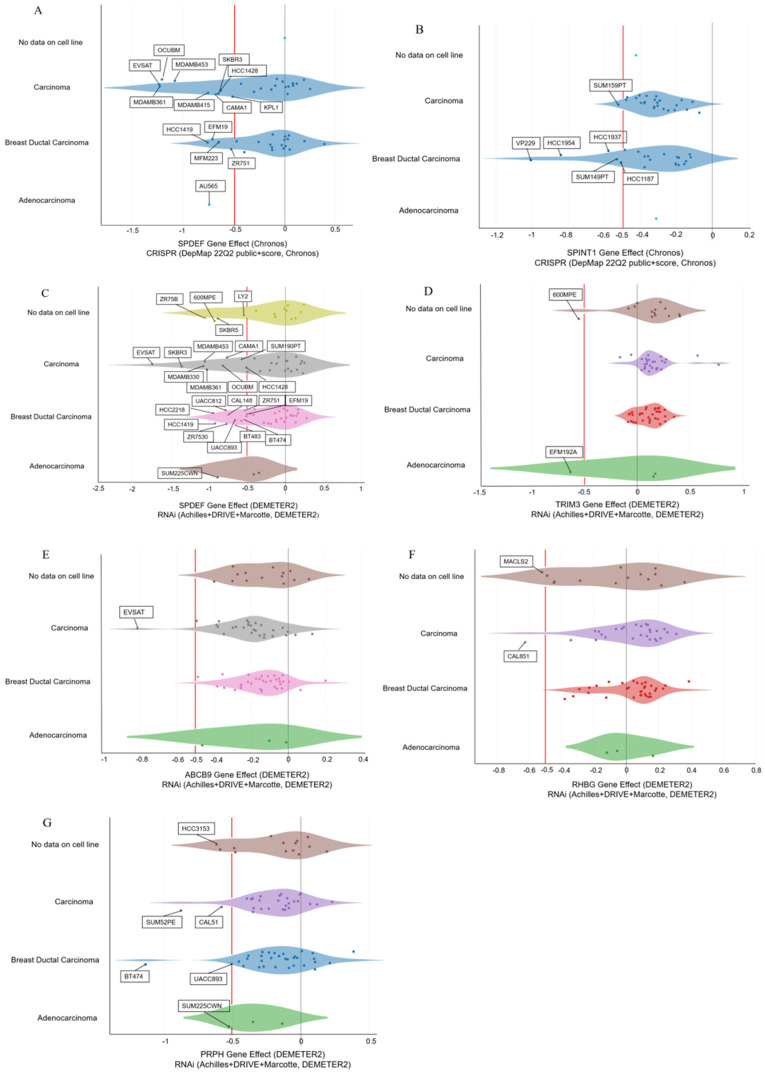
DepMap screening of candidate genes. Red line represents the cut-off value of −0.5. CRISPR-Cas9 screening: (**A**) *SPDEF* and (**B**) *SPINT1*. RNAi screening: (**C**) *SPDEF*, (**D**) *TRIM3*, (**E**) *ABCB9*, (**F**) *RHBG*, and (**G**) *PRPH*.

We next examined the gene effect scores of the nine genes in BC cell lines using DepMap. In *SPDEF*, 14 cell lines were below the cut-off value (−0.5) of the gene effect score for CRISPR-Cas9 data (14/27, 29.8%; Figure 7A). In *SPINT1,* six cell lines were below the cut-off value (−0.5) of the gene effect score for CRISPR-Cas9 data (6/47, 12.8%, Figure 7B). In *SPDEF*, 24 cell lines were below the cut-off value (−0.5) of the gene effect score for RNAi data (24/77, 31.2%, Figure 7C). In *TRIM3*, two cell lines were below the cut-off value (−0.5) of the gene effect score for RNAi data (2/73, 2.7%, Figure 7D). In *ABCB9*, one cell line was below the cut-off value (−0.5) of the gene effect score for RNAi data (1/78, 1.3%, Figure 7E). In *RHBG*, two cell lines were below the cut-off value (−0.5) of the gene effect score for RNAi data (2/75, 2.7%, Figure 7F). In *PRPH*, six cell lines were below the cut-off value (−0.5) of the gene effect score for RNAi data (6/76, 7.9%, Figure 7G). The details of the scores and cell lines are presented in Table 2 and Table 3, respectively. CRISPR-Cas9 screening results for *TRIM3*, *ABCB9*, *HSPB1*, *RHBG*, *EPN3*, *LRFN2*, and *PRPH* as well as RNAi screening results for *HSPB1*, *SPINT1*, and *LRFN2* are summarized in Supplementary Materials.

## 4. Discussion

In this study, we used LinkedOmics to extract a set of genes involved in the T and N stages of BC progression and identified nine genes with overlapping expression. We examined their expression levels between normal breast tissues and BC tumor tissues and among intrinsic subtypes of BC using UALCAN and applicability as potential therapeutic targets in BC using DepMap.

LinkedOmics analysis revealed that *SPDEF*, *TRIM3*, *ABCB9*, *HSPB1*, *RHBG*, *SPINT1*, *EPN3*, *LRFN2*, and *PRPH* were involved in both T and N stages of BC. In UALCAN analysis, *SPDEF*, *TRIM3*, *ABCB9*, *HSPB1*, *RHBG*, *SPINT1*, *EPN3*, and *LRFN2* were found to be highly expressed in BRCA tissues (Figure 4, *p* < 0.01). However, *PRPH* expression was lower than that in normal tissues (Figure 4I, *p* = 0.876). In the present study, *RHBG*, *LRFN2*, and *PRPH* were also found to be involved in the progression of BC. The relationship between *RHBG* and BC has not been reported to date; this study is the first to report that *RHBG* is directly involved in the progression of BC. *RHBG* has been shown to be a direct target of β-catenin regulation [21]. Further, since it has been shown to affect Wnt/β-catenin signaling for immune microenvironment regulation, stem cell maintenance, therapeutic resistance, and phenotyping [22], *RHBG* may be involved in BC progression via β-catenin. Regarding the relationship between *LRFN2* and cancer, *LRFN2* is known to inhibit esophageal cancer progression via the regulation of the Wnt/β-catenin and NF-κB signaling pathways [23]. Its relevance to BC remains unclear, as in esophageal cancer, *LRFN2* may be involved in BC progression via the Wnt/β-catenin and NF-κB signaling pathways. Further studies on the relationship between these genes and BC should be carried out in the future. *PRPH* encodes a 58 kDa type III intermediate protein filament that is mainly found in the peripheral and central nervous system neurons that project to peripheral structures, such as lower motor neurons that assemble with neurofilaments [24]. In recent years, it has been shown that the autonomic nervous system can reach the BC tissue, significantly impacting BC growth and metastasis [25]. As mentioned above, *PRPH* is known to associate with peripheral nerve formation [24], *LRFN2* is known to recruit and combine with the N-methyl-d-aspartate receptor (NMDARs) through the C-terminal PDZ domain to promote neurodevelopment [26,27,28], and *PRPH* and *LRFN2* may also be involved in this mechanism. Therefore, the expression of *LRFN2* and *PRPH* may be stepwise upregulated with the progression of BC.

We validated the expression of nine genes among intrinsic subtypes using UALCAN. We found that *SPDEF*, *TRIM3*, *HSPB1*, *RHBG*, *EPN3*, and *LRFN2* were significantly highly expressed in the luminal type than those in TNBC (Figure 5, *p* < 0.01); the luminal type was reported to have a considerably higher frequency of positive lymph nodes than TNBC [29]. In this study, because we extracted genes that are upregulated in the N stage, these genes are thought to be highly expressed in the luminal type. High *SPDEF* expression was reported to be more common in the luminal type and is positively correlated with the TNM stage, lymphoid nodal status, and tumor invasion, with a poor prognosis in high-expression groups [30,31,32]. *TRIM3* promotes estrogen signaling and BC progression [33]. The results of these previous studies are consistent with those of the present study. *SPDEF*, *TRIM3*, *HSPB1*, *SPINT1*, *EPN3*, and *LRFN2* were significantly highly expressed in the HER2-positive type than in TNBC (Figure 5, *p* < 0.05), as the HER2-positive type is reported to have a larger tumor size and higher stage [34,35]. We extracted genes that were upregulated and whose expression overlapped between the T and N stages; it is thought that genes related to tumor size, cell proliferation, and higher stage were highly expressed in the HER2-positive type. *HSPB1* is frequently upregulated in BC [36], wherein it promotes cancer cell growth and metastasis by inducing the SUMOylation of *HSPB8* and increasing its expression [37], and *SPINT1* was highly expressed in the HER2-positive type, with a relatively high rate in node-positive patients [38]. The results of these previous studies are consistent with those of the present study. *PRPH* was the only gene whose expression was lower in BC tissues than in normal tissues. Its expression level in the TNBC type was significantly higher than that in the HER2-positive type (Figure 5I, *p* = 0.0103) and demonstrated the difference in the distribution of intrinsic subtypes from the other eight genes.

Prognostic analysis by GEPIA2 showed that the high-expression group of *ABCB9* and *SPINT1* had a significantly worse prognosis (*p* = 0.0034 and *p* = 0.0067, respectively), whereas prognosis for the other genes, although involved in BC progression, did not differ remarkably between the high and low expression groups. Genes involved in BC progression were not considered to equate with poor prognostic factors. *ABCB9* is highly expressed in paclitaxel-resistant BC, and microRNA-24 is known to suppress *ABCB9* and increase paclitaxel sensitivity [39]. For *SPINT1*, KEGG enrichment analysis showed that *ErbB* signaling is substantially enriched. *ErbB* signaling is involved in regulating drug resistance [40,41]. Because the GEPIA2 dataset includes patients with a history of drug treatment, it is likely that the prognosis differed according to the genes involved in drug resistance.

In this study, we identified a set of genes involved in BC progression, and DepMap analysis was used to validate the dependency and essentiality of the nine genes for proliferation and survival of BC cell lines and whether they are potential therapeutic targets. Regarding *SPDEF*, *TRIM3*, *ABCB9*, *RHBG*, *SPINT1*, and *PRPH*, the cell lines below the cut-off value (gene effect score: −0.5) were a part of the total (1.3–31.2%). These genes proved essential for BC cell line survival; knocking out or knocking down their expression has been shown to serve as a potential therapeutic targeting approach. As *SPDEF*, *TRIM3*, *ABCB9*, and *SPINT1* have already been linked to BC, knocking out or knocking down the expression of these genes can suppress the growth of or kill BC cell lines. Interestingly, the finding that PRPH, a neuronal intermediate filament protein, is essential for survival in several BC cell lines is novel. We initially thought that since PRPH is a type III intermediate filament protein expressed mainly in the neurons of the peripheral nervous system, its elevated expression was the result of the upregulation of an upstream transcription factor, rather than *PRPH* being associated with BC. However, when the knockdown of *PRPH* was performed using RNAi, 7.9% of cell lines were below the cut-off value (−0.5), suggesting a direct causal relationship between *PRPH* and BC. The results of this study are of high clinical significance as *PRPH* was found to be a potential therapeutic target in BC. 

Additional studies are needed to elucidate the role of *PRPH* in BC progression and survival and the underlying molecular mechanisms. Originally, knockout of a gene using CRISPR-Cas9 was considered to have a more serious impact than knockdown using RNAi. However, it is now clear that knockdown of a gene has a greater effect than its knockout [42]. A genetic compensation response is said to activate the transcription of genes related to the gene inactivated by knockout. In other words, the expression of genes associated with the knockout gene increases, complementing the knocked-out gene function, often having no effect on the cell. However, this mechanism does not occur when the expression of a gene is knocked down, and as a result, several genes that are knocked down by RNAi can induce biological defects [43]. This mechanism may explain why RNAi had a more severe effect on several cell lines in this study; 1.3%–31.2% of the total cell lines were below the cut-off value (−0.5) in the cases of *SPDEF*, *SPINT1*, *TRIM3*, *ABCB9*, *RHBG,* and *PRPH.* Currently, there are no molecular therapies targeting *SPDEF*, *SPINT1*, *TRIM3*, *ABCB9*, *RHBG*, or *PRPH*. However, since the current study showed that knockout/knockdown of these genes was essential for the survival of BC cell lines, development of drugs targeting these genes might be worth exploring in the future. To establish novel therapeutic strategies for BC, it is essential to conduct active translational research involving previously reported genes (i.e., *LXR alpha*, *SEMA6D*, and *ITGA7*) and those identified in the current study. 

Our study has some limitations. This study was conducted using bioinformatics only; hence, additional evaluation, validation, verification, and demonstration with actual BC tissues and cell lines are required. In addition, screening with the DepMap database successfully identified genes that could be potential therapeutic targets; however, a more careful analysis is needed if these gene groups are to be considered as drug target candidates. Whether these genes are prognostic factors should be stringently established and precisely matched to treatment history, treatment method, and staging.

In summary, *SPDEF*, *TRIM3*, *ABCB9*, *HSPB1*, *RHBG*, *SPINT1*, *EPN3*, *LRFN2*, and *PRPH* are involved in BC progression. *ABCB9* and *SPINT1* are associated with worse prognosis in BC. Furthermore, *SPDEF*, *TRIM3*, *ABCB9*, *RHBG*, *SPINT1*, and *PRPH* were found to be essential for survival in some BC cell lines, indicating that these genes are potential therapeutic targets in BC. We hope that future studies will verify these findings using BC tissues and cell lines and validate these genes as new and effective therapeutic targets.

## Figures and Tables

**Table 1:** Upregulated genes with overlapping expression in T and N stages.

Gene ID	Gene Symbol	Gene Name	*p*-Value (T Stage)	*p*-Value (N Stage)
25,803	*SPDEF*	SAM pointed domain-containing ETS transcription factor	6.28 × 10^−4^	7.07 × 10^−6^
10,612	*TRIM3*	Tripartite motif containing 3	3.96 × 10^−3^	3.05 × 10^−5^
23,457	*ABCB9*	ATP binding cassette subfamily B member 9	7.00 × 10^−5^	3.35 × 10^−5^
3315	*HSPB1*	Heat shock protein family B (small) member 1	4.35 × 10^−3^	3.91 × 10^−5^
57,127	*RHBG*	Rh family B glycoprotein	6.17 × 10^−4^	9.17 × 10^−5^
6692	*SPINT1*	Serine peptidase inhibitor, Kunitz type 1	2.39 × 10^−3^	1.82 × 10^−4^
55,040	*EPN3*	Epsin 3	1.56 × 10^−3^	2.72 × 10^−4^
57,497	*LRFN2*	Leucine rich repeat and fibronectin type III domain-containing 2	4.02 × 10^−3^	4.11 × 10^−4^
5630	*PRPH*	Peripherin	5.85 × 10^−4^	8.92 × 10^−4^

**Table 2:** Gene effect score of CRISPR (DepMap 22Q1 public + score, Chronos).

Gene Symbol	Cell Line	Subtype	Gene Effect Score
*SPDEF*	EVSAT	Unknown	−1.24
	MDAMB361	HER2	−1.23
	OCUBM	Unknown	−1.21
	MDAMB453	HER2	−1.08
	MDAMB415	HER2	−0.76
	HCC1419	HER2	−0.76
	AU565	HER2	−0.74
	EFM19	Luminal	−0.71
	CAMA1	Luminal	−0.68
	SKBR3	HER2	−0.66
	MFM223	Luminal	−0.65
	HCC1428	Luminal	−0.63
	ZR751	Luminal	−0.53
	KPL1	Luminal	−0.52
*SPINT1*	VP228	Unknown	−1.00
	HCC1954	TN	−0.84
	HCC1937	TN	−0.58
	SUM149PT	TN	−0.53
	SUM159PT	TN	−0.52
	HCC1187	TN	−0.51

TN: triple negative.

**Table 3:** Gene effect scores of RNAi (Achilles + DRIVE + Marcotte, DEMETER2).

Gene Symbol	Cell Line	Subtype	Gene Effect Score	Cell Line	Subtype	Gene Effect Score
*SPDEF*	EVSAT	Unknown	−1.74	SKBR3	HER2	−1.35
	MDAMB330	Unknown	−1.07	MDAMB453	HER2	−1.05
	ZR75B	Unknown	−1.05	MDAMB361	HER2	−1.03
	600MPE	Unknown	−0.94	HCC228	HER2	−0.94
	HCC1419	HER2	−0.92	SKBR5	Unknown	−0.88
	SUM225CWN	TN	−0.88	OCUMBM	Unknown	−0.82
	UACC812	Luminal	−0.78	ZR7530	HER2	−0.77
	CAMA1	Luminal	−0.76	CAL148	TN	−0.74
	UACC893	HER2	−0.66	BT483	Luminal	−0.65
	BT474	HER2	−0.58	SUM190PT	TN	−0.57
	LY2	Unknown	−0.54	ZR751	Luminal	−0.53
	HCC1428	Luminal	−0.51	EFM19	Luminal	−0.51
*TRIM3*	EFM192A	HER2	−0.63	600MPE	Unknown	−0.55
*ABCB9*	EVSAT	Unknown	−0.81			
*RHBG*	CAL851	TN	−0.63	MACLS2	Unknown	−0.52
*PRPH*	BT474	HER2	−1.13	SUM52PE	Luminal	−0.88
	HCC3153	Unknown	−0.62	CAL51	TN	−0.58
	SUM225CWN	TN	−0.53	UACC893	HER2	−0.51

TN: triple negative.

## Data Availability

The datasets generated and/or analyzed during this study are available from the corresponding author upon reasonable request.

## Supplementary Materials

The following supporting information can be downloaded at: https://www.mdpi.com/article/10.3390/biomedicines10112995/s1, Figure S1: CRISPR-Cas9 screening: (a) *TRIM3*, (b) *ABCB9*, (c) *HSPB1*, (d) *EPN3*, (e) *LRFN2*, and (f) *PRPH*. RNAi screening: (g) *HSPB1*, (h) *SPINT1*, and (i) *LRFN2*; Table S1: Statistical comparison of normal, luminal, HER2, and TNBC type for the nine candidate genes.
