# Supplementary material for: In Silico Identification of Genes Associated with Breast Cancer Progression and Prognosis and Novel Therapeutic Targets
Source: Biomedicines. 2022 Nov 21;10(11):2995. doi: 10.3390/biomedicines10112995 (PMC9687996; doi:10.3390/biomedicines10112995)
Supplement: Supplementary file 1 [file biomedicines-10-02995-s001.zip › biomedicines-1987789-table S1.pdf]

Additional files: Supplemental Table S1

Gene	Comparison	Stastical significance
SPDEF	Normal vs Luminal	1.62E-12
	Normal vs HER2	2.28E-06
	Normal vs TNBC	4.82E-04
	Luminal vs HER2	8.42E-01
	Luminal vs TNBC	1.62E-12
	HER2 vs TNBC	4.14E-08
TRIM3	Normal vs Luminal	1.11E-16
	Normal vs HER2	9.79E-04
	Normal vs TNBC	1.89E-06
	Luminal vs HER2	8.50E-02
	Luminal vs TNBC	<1E-12
	HER2 vs TNBC	1.9E-05
ABCB9	Normal vs Luminal	1.62E-12
	Normal vs HER2	6.05E-05
	Normal vs TNBC	5.38E-09
	Luminal vs HER2	2,19E-02
	Luminal vs TNBC	4.24E-01
	HER2 vs TNBC	5.75E-02
HSPB1	Normal vs Luminal	1.62E-12
	Normal vs HER2	5E-03
	Normal vs TNBC	1.9E-02
	Luminal vs HER2	2.27E-07
	Luminal vs TNBC	2.69E-07
	HER2 vs TNBC	9.78E-01
RHBG	Normal vs Luminal	1.11E-16
	Normal vs HER2	6.35E-02
	Normal vs TNBC	3.59E-05
	Luminal vs HER2	3.95E-02
	Luminal vs TNBC	7.26E-09
	HER2 vs TNBC	6.03E-01
SPINT1	Normal vs Luminal	1.62E-12
	Normal vs HER2	1.44E-15
	Normal vs TNBC	2.92E-10

	Luminal vs HER2	1.17E-01
	Luminal vs TNBC	3.88E-01
	HER2 vs TNBC	4.24E-02
EPN3	Normal vs Luminal	1.62E-12
	Normal vs HER2	1.24E-03
	Normal vs TNBC	5.67E-07
	Luminal vs HER2	7.24E-02
	Luminal vs TNBC	<1E-12
	HER2 vs TNBC	3.58E-03
LRFN2	Normal vs Luminal	1.62E-12
	Normal vs HER2	4.36E-04
	Normal vs TNBC	1.09E-02
	Luminal vs HER2	6.41E-01
	Luminal vs TNBC	1.74E-12
	HER2 vs TNBC	4.68E-03
PRPH	Normal vs Luminal	7.48E-01
	Normal vs HER2	1.62E-12
	Normal vs TNBC	9.75E-01
	Luminal vs HER2	7.55E-02
	Luminal vs TNBC	9.75E-01
	HER2 vs TNBC	1.03E-02
