# Supplementary figures and images for: In Silico Identification of Genes Associated with Breast Cancer Progression and Prognosis and Novel Therapeutic Targets
Source: Biomedicines. 2022 Nov 21;10(11):2995. doi: 10.3390/biomedicines10112995 (PMC9687996; doi:10.3390/biomedicines10112995)

Fig.S1A

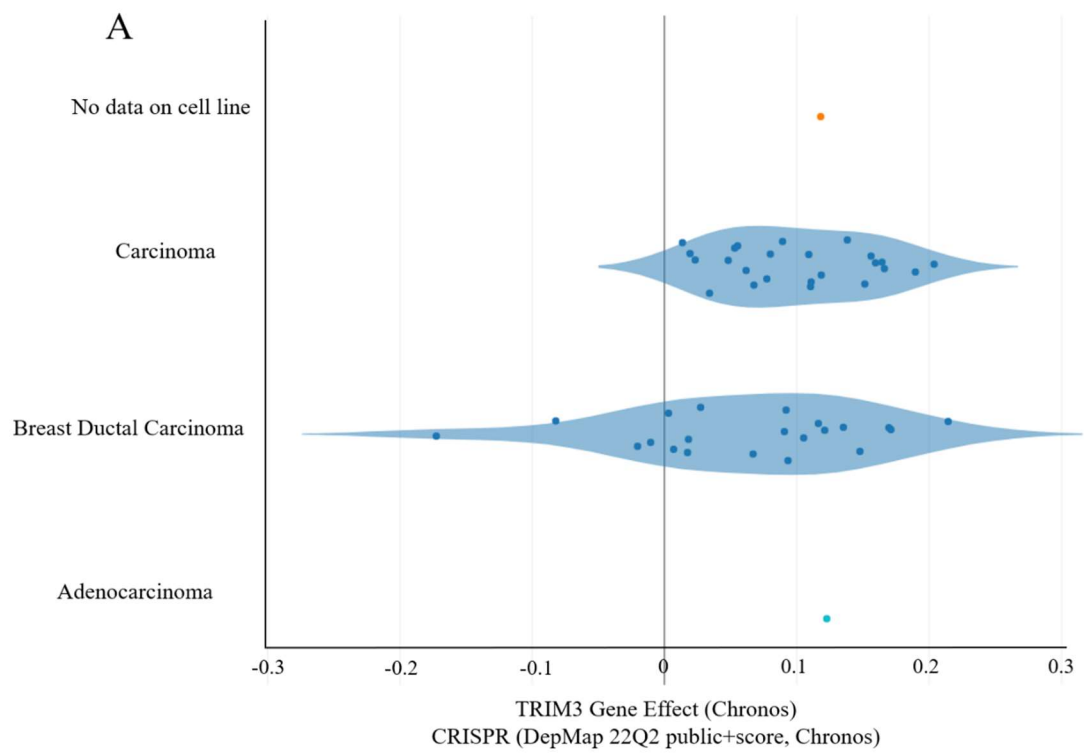

Fig.S1B

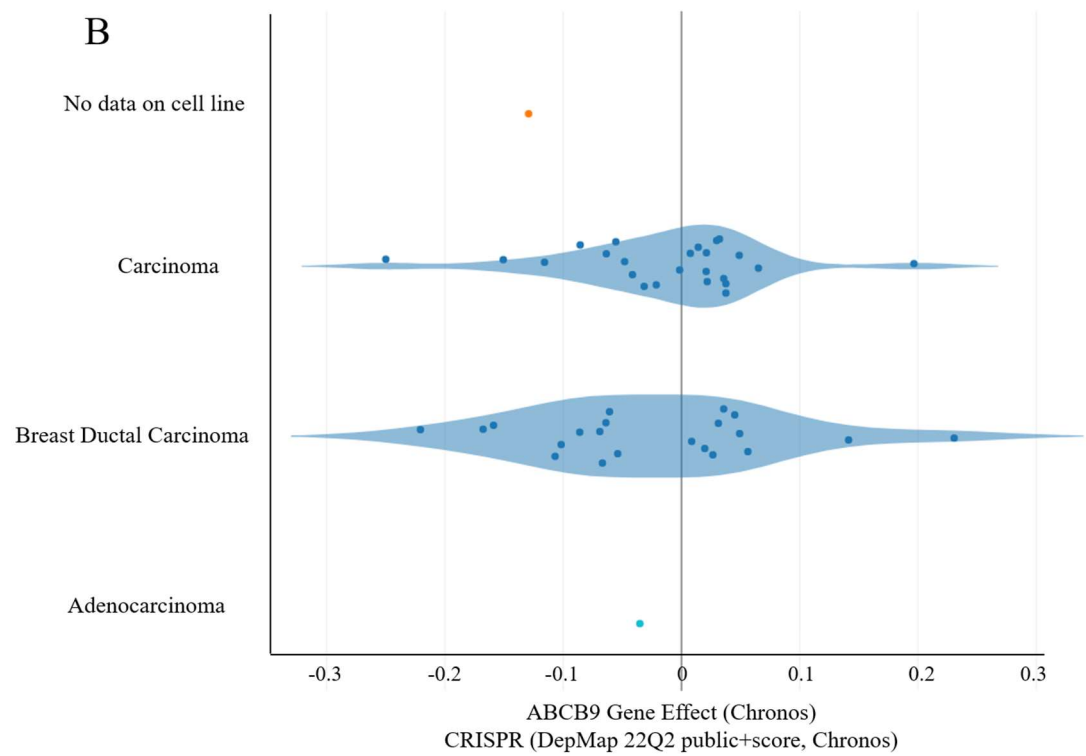

Fig.S1C

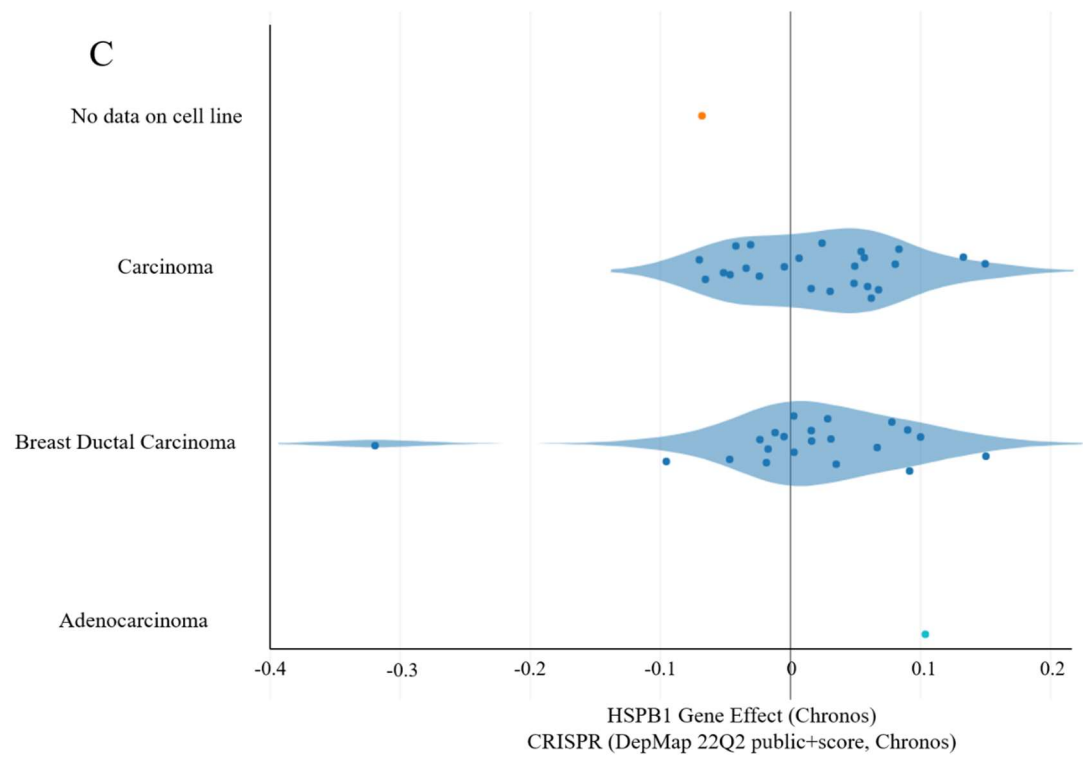

Fig.S1D

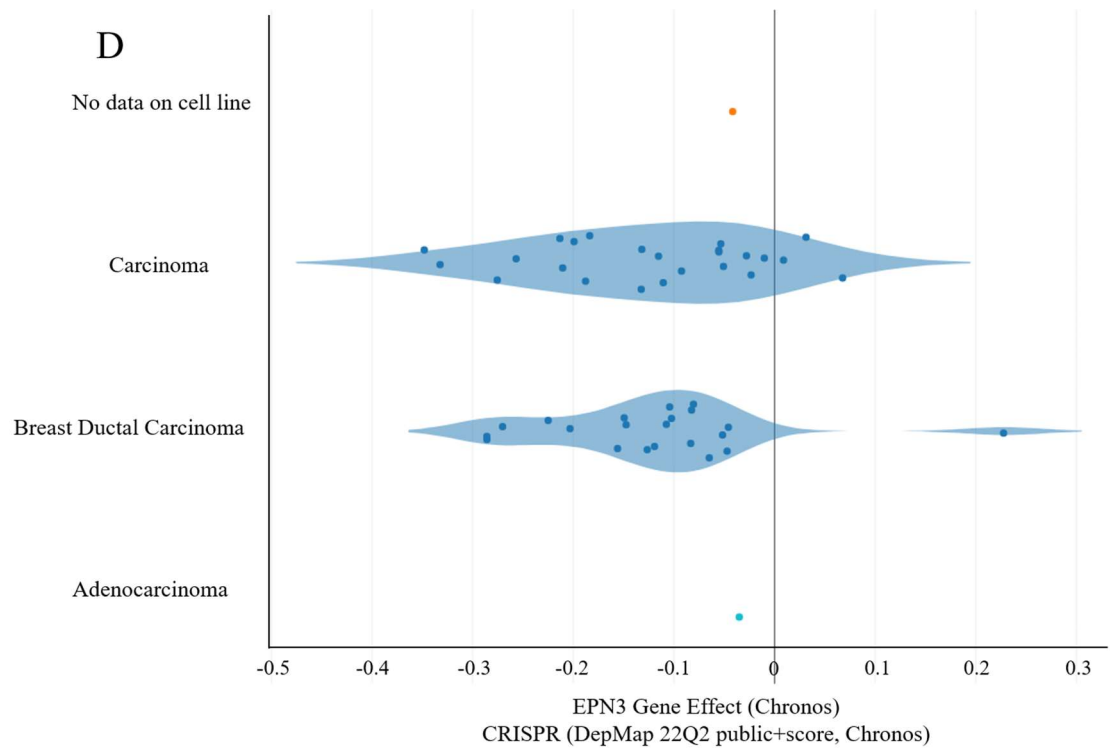

Fig.S1E

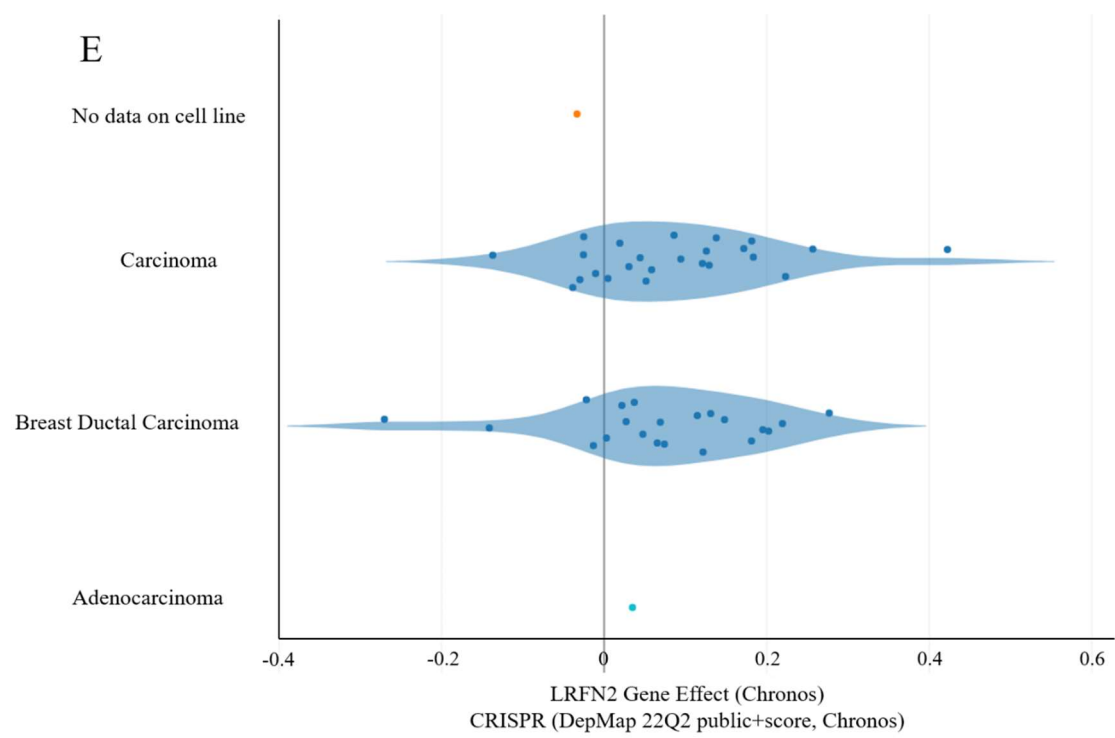

Fig.S1F

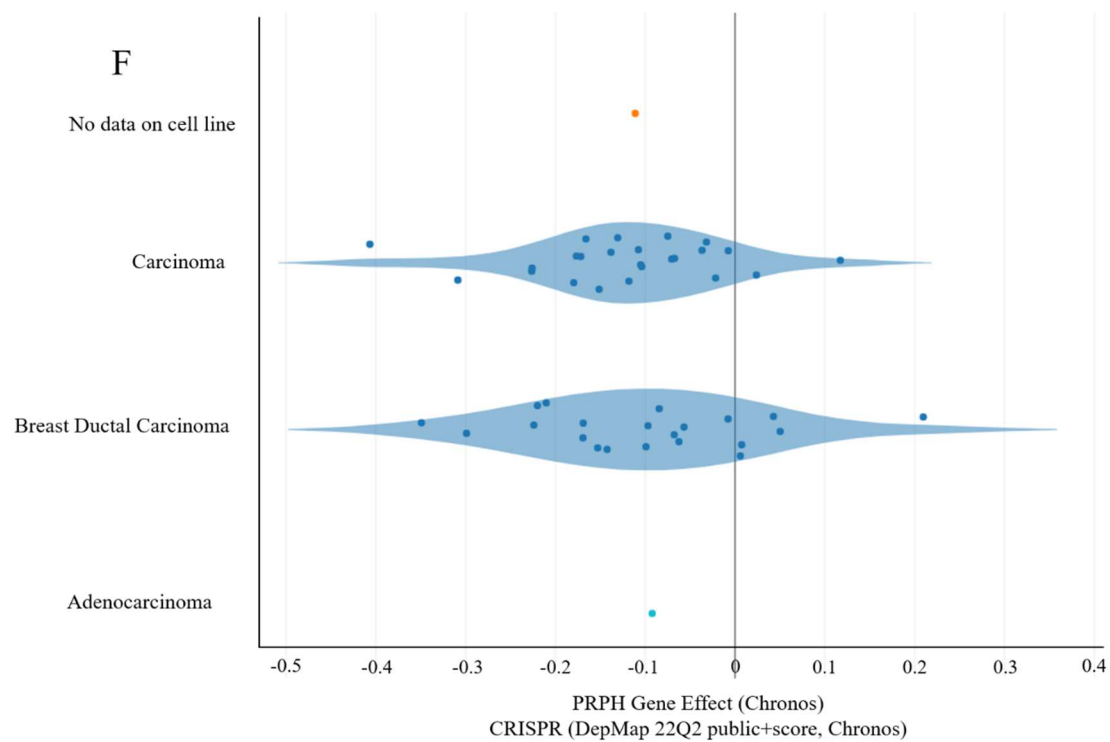

Fig.S1G

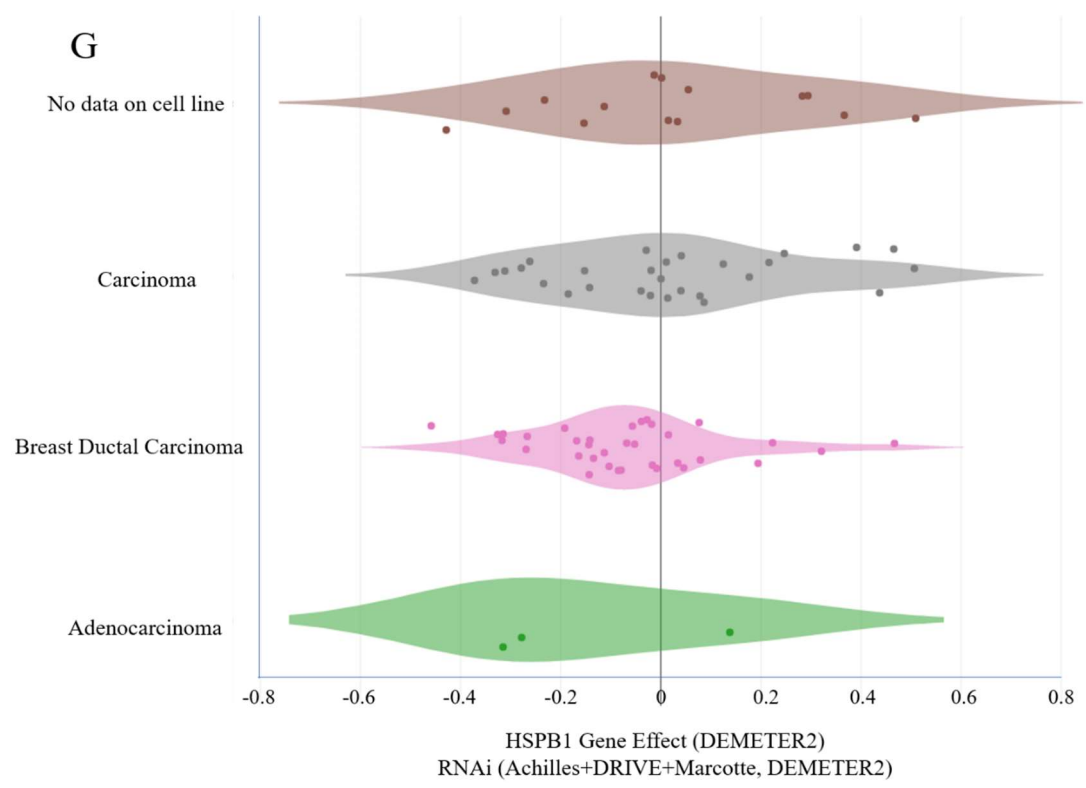

Fig.S1H

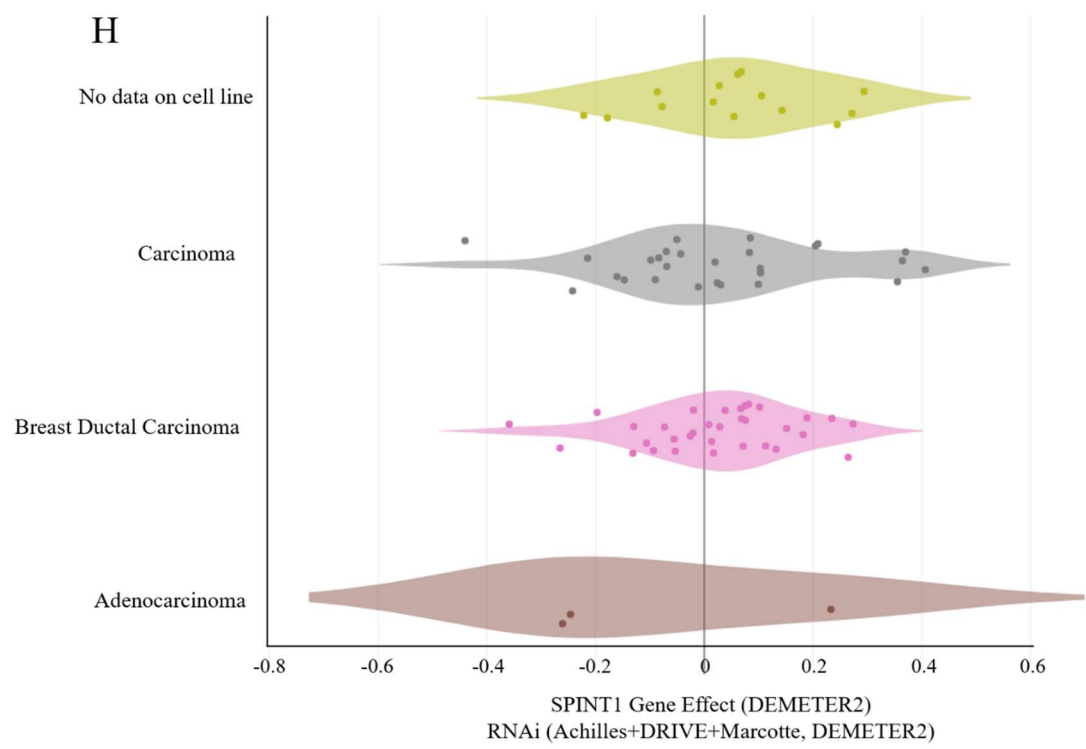

Fig.S1I

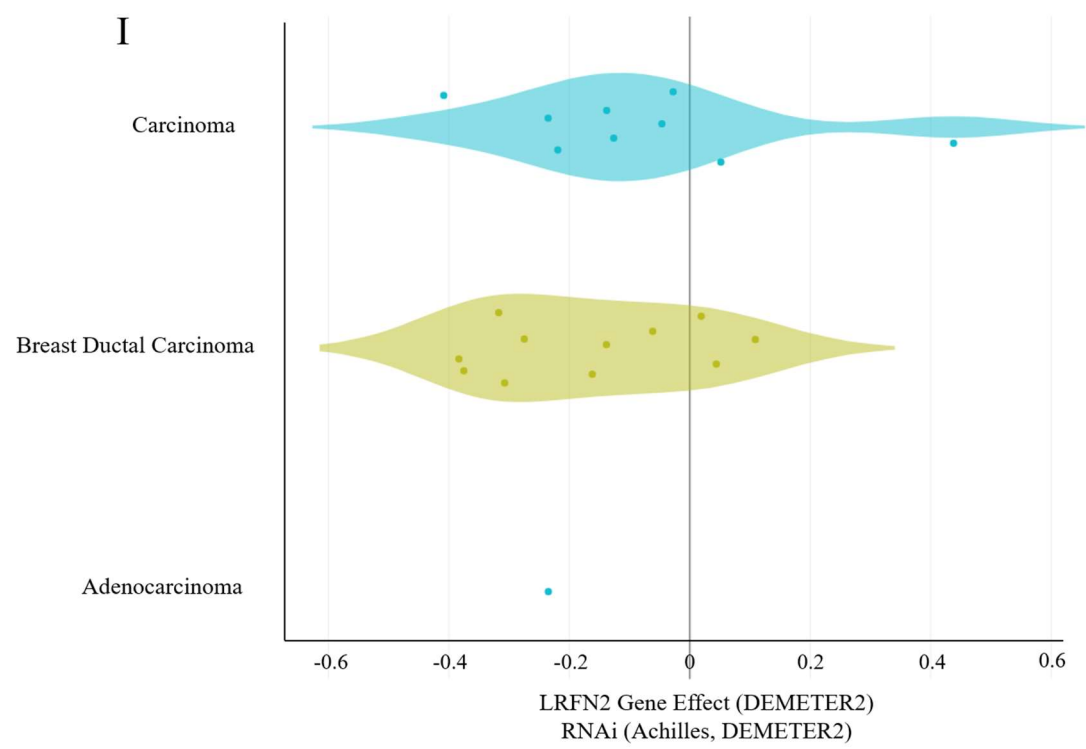

Supplement: Supplementary file 1 [file biomedicines-10-02995-s001.zip › biomedicines-1987789-figure S1.pdf]
